# Association of dietary patterns, number of daily meals and anthropometric measures in women in age of menopause

**DOI:** 10.20945/2359-3997000000414

**Published:** 2021-11-11

**Authors:** Érika Brombil França, Karina Giane Mendes, Heloísa Theodoro, Maria Teresa Anselmo Olinto, Raquel Canuto

**Affiliations:** 1 Universidade Federal do Rio Grande do Sul Programa de Pós-graduação em Alimentação, Nutrição e Saúde Porto Alegre RS Brasil Programa de Pós-graduação em Alimentação, Nutrição e Saúde, Universidade Federal do Rio Grande do Sul, Porto Alegre, RS, Brasil; 2 Universidade de Caxias do Sul Centro de Biologia e Ciências da Saúde Caxias do Sul RS Brasil Centro de Biologia e Ciências da Saúde, Universidade de Caxias do Sul, Caxias do Sul, RS, Brasil; 3 Universidade do Vale do Rio dos Sinos Programa de Pós-graduação em Saúde Coletiva São Leopoldo RS Brasil Programa de Pós-graduação em Saúde Coletiva, Universidade do Vale do Rio dos Sinos, São Leopoldo, RS, Brasil; 4 Hospital de Clínicas de Porto Alegre Centro de Estudos em Alimentação e Nutrição (Cesan) Porto alegre RS Brasil Centro de Estudos em Alimentação e Nutrição (Cesan), Hospital de Clínicas de Porto Alegre (HCPA), Porto alegre, RS, Brasil

**Keywords:** Dietary patterns, women, waist circumference, body mass index, anthropometric measures, meals, obesity, abdominal obesity

## Abstract

**Objective:**

To investigate the association of dietary patterns, number of daily meals and anthropometric measures among women in age of menopause.

**Subjects and methods:**

This was a transversal study with 320 women over 50 years old from Caxias do Sul, Brazil. The outcomes were body mass index (BMI) and waist circumference (WC). Multiple linear regression was performed.

**Results:**

Three dietary patterns: regional, fruits and vegetables, and common Brazilian was identified by Principal Component Analysis. After adjustment, higher adoption of the regional dietary pattern was associated with increased BMI (β = 0.56 [CI95% = 0.03-1.08], p = 0.037) and WC (β = 1.28 [CI95% = 0.17-2,55], p = 0.047). The highest number of meals per day (>=5/day) was associated with reduced BMI (β = -1.18 [CI95% = -2.30 to -0.05], p = 0.041) and WC (β = -2.77 [CI95% = -5.41 to -0.13], p = 0.039), and a mid-afternoon snack BMI (β = -2.16 [CI95% = -3.66 to -0.65], p = 0.005) and WC (β = -5.76 [CI95% = -9.29 to -2.23], p = 0,001). The regional dietary pattern was inversely associated with have five or more meals per day (β = -0.51 [CI95% = -0.84 to -0.18], p = 0.002) and have a mid-afternoon snack (β = -0.63 [CI95% = -1.07 to -0.18], p = 0.006). The fruit and vegetables dietary pattern was positively associated with have five or more meals per day (β = 0.35 [CI95% = 0.02-0.69], p = 0.034).

**Conclusion:**

The regional dietary pattern has resulted in higher BMI and WC measures and contributes to decreased meals per day, behavior associated with higher anthropometric measures.

## INTRODUCTION

The occurrence of excess weight increases with age, especially in women (1,2) and leads to an increased risk of morbidity and mortality, prejudice to the quality of life, and complications that may significantly influence life expectancy (3). Among the risk factors for the development of obesity in women with advancing age, menopause can be highlighted as a period of hormonal changes that generate biological, endocrine and clinical changes followed by symptoms that result in poor quality of a woman’s life and predisposes the accumulation of body fat (4).

Studies have shown positive changes in women’s eating habits as they age, such as adopting healthier dietary patterns, including fruit and vegetable consumption (5). One of the main methods of evaluating eating habits in the population is identifying dietary patterns through *posteriori* statistical methods (6). Studies with this scope have suggested that dietary patterns considered healthy have a protective effect on health (7-10). On the other hand, unhealthy dietary patterns lead to excessive weight gain. Furthermore, foods that compose healthy and unhealthy dietary patterns vary significantly among the studies, showing cultural and sociodemographic differences in food consumption by different populations (7-10), which is evidence of the importance of investigating dietary patterns and their association with overall health in different populations. In addition, a recent systematic review of the literature regarding the association between dietary intake and menopausal symptoms found only three studies that had investigated women’s dietary patterns, and it was from China and Iran, demonstrating the need for more studies in this field (10).

Another aspect of food habits described as a possible risk factor for weight gain is eating frequency (the number of daily meals). Although there is a recommendation to divide food intake into a larger number of meals during the day to better control or lose weight (11), the studies that analyzed this association have presented different results so far (12,13). In this way, some studies have already found that the number of meals per day is related to diet quality (14,15). However, as far as we know, no study investigated whether different dietary patterns (identified through principal component analysis) are associated with food consumption frequency in adults (12).

Considering the importance of know the dietary patterns of Brazilian menopause women, the lack of studies on the association between dietary patterns and number of daily meals and the controversial evidence regarding the impact of eating frequency on body weight, this study aimed to identify dietary patterns and to investigate their association with BMI and waist circumference and the number of daily meals in Brazilian women in age of menopause. In addition, we also investigated the association between daily meals and anthropometric measures.

## SUBJECTS AND METHODS

It is a transversal analytical study conducted with a sample of women over 50 years old enrolled in the Coexist Project program in Caxias do Sul (RS). The Project offers to the community continuous activities focusing on leisure physical activity, and biological, social, emotional and spiritual aspects. The activities are organized and guided by Physical Education professionals.

Data collection was done along the years 2014 and 2015. A random sample of 10% of the women included in the study was used for quality control to evaluate the research’s internal validity. In addition, a simplified questionnaire was administered by telephone. The study was approved by the Ethics Committee at the University of Caxias do Sul (Registry no. CAEE 17990213.9.0000.5321). The participates received all information through a Free and Informed Consent Form.

The size of the sample needed to estimate the prevalence of abdominal obesity was calculated using the following parameters: (1) number of women over 50 years old living in Caxias do Sul (2010 Census): 53,093; (2) confidence interval of 95%; (3) estimated prevalence of abdominal obesity on women over 50 years-old: 66.6%; (4) confidence limit of 4 p.p. (62 to 70%). This resulted in a sample size of 340 women. We have followed the rule proposed by Hair and cols. (2009) to study dietary patterns (16).

Project Coexist in Caxias do Sul is formed by 72 groups, with around 40 women each, 90% of them over 50 years old. The selection of the participants was made by systematic sampling in multiple stages. First, the 72 groups were numbered according to their geographic location; then, the total number of groups (72) was divided by the number of necessary groups (16), which resulted in 4.5 (rounded up to 4). Finally, the number 2 was drafted (among numbers 1 to 4) and became the starting point for selecting the groups.

For the next stage, all the women over 50 who participated in the 16 selected groups were invited to participate. Women who have physical problems that difficult to take anthropometric measures or show cognitive difficulties to answer the questionnaire were excluded. All the women who accepted to participate and fulfilled the eligibility criteria were included. On average, 20 women per group were included, totaling 320 women participants. We had ten recuses in the process.

Information on economic, demographic, behavioral, anthropometric and food consumption characteristics was obtained through standardized questionnaires, pre-coded and pre-tested, carried out by previously trained interviewers.

The anthropometric variables were BMI and WC. Weight was measured by a portable digital scale (Plenna) with a capacity of up to 150 kg. Women were weighed, standing up, barefoot, with minimal clothing, with their arms resting alongside their bodies. When measuring height, women had to be standing up, barefoot, with minimal clothing, with their arms resting alongside their bodies and looking upfront. Height measurements were taken using a vertical anthropometer, fixed on a smooth wall with no skirting board, positioned to provide an accurate height measure. BMI was calculated by the weight/height ratio in kg/m². BMI was analyzed as a continuous variable (17).

WC was measured in centimeters at the medium point between the iliac crest and the inferior costal margin (18). Measurements were taken twice, and the mean value of these two measurements was used. WC was analyzed as a continuous variable.

Dietary patterns were determined using a food frequency questionnaire (FFQ) validated for the adult population of Porto Alegre, Brazil (19), made up of 65 foods and the amount and frequency they are consumed, as well as the number of times (0 to 7) they are consumed daily, weekly, monthly in the last year. Another ten food items typically consumed by the population in this study were included. The consumption of the food items was transformed into annual consumption frequency, and for seasonal items, the frequency was divided by four (the four seasons of the year).

The number of daily meals was measured by a standardized questionnaire that investigated the habitual consumption of the following meals: breakfast (6-7 a.m.), mid-morning snack (9-10 a.m.), lunch (12-1 p.m.), mid-afternoon snack (4-5 p.m.), dinner (8-9 p.m.), evening snack (11 p.m.-12 a.m.), middle of the night snack (3-4 a.m.), and, also, whether they had any other snacks between meals. The total number of meals in a day (≤ 4 meals per day or ≥ 5 meals per day) and each isolated meal was analyzed.

Demographic and socioeconomic characteristics investigated were: age, measured by full years and categorized as such: 50 to 60; 61 to 70; 71 or more; marital status: married, single, widowed, divorced; skin color/ethnicity: white or non-white; education: none, one to five years of education, six to eight years, nine years or more; and, family income measured by the number of minimum wage salaries earned: 0-2 salaries, 2.01-5 salaries, more than 5 salaries. Behavioral characteristics were: smoking (had smoked but quit, never smoked, or currently smokes); and the practice of at least 30 minutes of moderate physical activity: 0-2 times per week and 3 or more times per week.

### Statistical analysis

Data entry followed a double-entry protocol into EPI-DATA software (Denmark, version 3.1). Dietary patterns were identified through Principal Components Analysis (PCA). At first, 31 food groups were formed using these criteria: nutritional composition, positive and significant correlation between items p ≤ 0.05, as well as cultural aspects in their consumption.

After that, the applicability of the method was evaluated using Kaiser-Meyer-Olkin (The KMO ranges from 0 to 1; values below 0.5 are considered unacceptable, 0.50-0.59 miserable, 0.60-0.69 mediocre, 0.70-0.79 middling, 0.80-0.89 meritorious, and 0.90-1.0 marvelous) (20) and Bartlett tests (<0,001). To identify the factors to be retained, Kaiser Criteria were used, with eigenvalues over 1.0. The graphic of eigenvalues for each factor was used (scree plot) and the factors themselves to establish which series of factors most significantly described the patterns. An orthogonal rotation was done to maximize larger factorial loading and minimization of smaller loadings using Varimax rotation. The foods whose factor loadings were higher than 0.30 were kept in the matrix. The naming of each pattern, representing different dietary patterns, considered the foods that were a part of their patterns and the cultural aspects of their consumption. (16,21). Dietary patterns’ scores were analyzed as a continuous variable and into consumption quintiles.

The sample was described using absolute and relative frequencies. The mean values for BMI and WC were calculated for each variable, and associations with exposure variables were estimated by ANOVA and Student’s T-test. Multiple linear regression was performed to investigate the association between interest expositions (dietary patterns and the number of daily meals) and outcomes (BMI and WC). Variables that presented a significance level of 20% (p < 0,20) in the simple linear analysis were considered potential confounders in the association between main exposure variables and outcomes. The association between meals per day (type and number of meals) and dietary patterns was explored using simple linear regression analysis. In all the association analyses, a significance level of 5% was taken into consideration. The analyses were done using STATA version 12.0 (StataCorp., CollegeStation, USA).

## RESULTS

The final sample was 320 women. A *posteriori* sample calculation (Alpha of 0.05 and beta of 0.20) showed that the sample of 320 women had power for demonstrating mean differences around 1.5 kg/m^2^ for dietary patterns and eating frequency with BMI; 4 cm for dietary patterns and WC; and 3.5 cm for eating frequency and WC.

The mean age was 67 years (SD = 8.32), mean BMI was 29.09 kg/m² (SD = 4.77) and WC 89.12 cm (SD = 11.31). Most of them were married, white/Caucasian, had less than 6 years of formal education and family income of less than 5 minimum wage salaries (73.1%). Most of them were non-smokers, and only 36% of the women practiced physical activity three or more times each week. There is a significant association between marital status and WC; widowed women had higher mean values for WC (Table 1).

**Table 1:** Mean, and standard deviation of body mass index and waist circumference according to the sociodemographic and behavioral characteristics of menopausal women of Caxias do Sul, Brazil

Variables		n (%)	BMI Mean (sd)	*p-value*	WC Mean (sd)	*p-value*
Age (years)				0.369		0.906
	50-60	77 (24.1)	29.68 (5.23)		89.32 (12.36)	
	61 a 70	135 (42.2)	29.11 (4.45)		88.80 (10.09)	
	≥71	108 (33.8)	28.66 (4.83)		89.40 (12.09)	
Marital status				0.195		0.034
	Not married	50 (15.6)	28.08 (3.38)		85.75 (9.98)	
	Married	166 (51.9)	29.10 (5.05)		89.10 (11.73)	
	Widowed	104 (32.5)	29.57 (4.86)		90.81 (10.97)	
Skin color				0.568		0.653
	White	272 (85.0)	29.02 (4.72)		89.00 (11.42)	
	Not white	47 (14.7)	29.46 (5.31)		89.20 (10.91)	
Education				0.493		0.507
	Not educated	6 (1.9)	26.78 (5.83)		84.16 (16.56)	
	1-5 years	168 (52.5)	28.90 (4.69)		89.64 (11.64)	
	6-8 years	56 (17.5)	29.62 (5.02)		87.73 (10.42)	
	≥9 years	89 (27.8)	29.23 (4.73)		89.24 (10.88)	
Family income				0.818		0.885
	0 a 2 MG	88 (27.5)	29.30 (4.92)		89.75 (11.27)	
	2,01 a 5 MG	146 (45.6)	28.96 (4.73)		89.09 (10.97)	
	>5,01 MG	80 (25.0)	29.31 (4.79)		88.98 (12.14)	
Physical Activity				0.331		0.085
	0-2 times a week	205 (64.1)	29.29 (4.84)		89.94 (11.42)	
	≥3 times a week	115 (35.9)	28.74 (4.65)		87.65 (11.02)	
Smoking				0.541		0.397
	Never	247 (77.2)	29.15 (4.80)		89.30 (11.46)	
	Former	62 (19.4)	29.15 (4.36)		88.95 (10.32)	
	Current	11 (3.4)	27.52 (6.44)		86.22 (13.86)	

*p-value* – ANOVA test: age, marital status, education, family income and smoking; t-test: skin color and physical activity; sd: standard deviation; BMI: body mass index; WC: waist circumference; MG: minimum wage.

Most women had five or more meals per day (67.5%), breakfast (96.9%) and mid-morning snacks (68.4%). All of them had lunch, most of them had a mid-afternoon snack (86.3%) and dinner (95.9%). Most women said they did not have an evening snack (57.8%) and a middle-of-the-night snack (95.9%) (data was not tabulated).

Three dietary patterns identified could be observed in Table 2. The first dietary pattern that better-explained food consumption in the sample was named Regional. It contained foods commonly consumed in the region these women lived, the Serra Gaúcha, such as white bread, jam, white rice and pasta, pumpkin, common regional fruits (grapes and tangerines) and fried polenta. The second dietary pattern was called Fruits and Vegetables because it contained mainly fruits and vegetables. The third eating pattern was called common Brazilian because it contained mainly foods that are commonly consumed in the Brazilian diet, such as cassava, chicken, eggs, fish, potatoes, black beans.

**Table 2:** Food items, factor loadings and explained variance for each dietary pattern

Dietary Pattern	Food	Factor loading	% of explained variance
DP I (Regional)	White bread (milk, french, homemade, sweet and *cuca*)	0.501	8.333
	Jam	0.436	
	White rice	0.416	
	Pasta	0.412	
	Pumpkin, carrot, beet and tomato	0.387	
	Soda (traditional and zero)	0.383	
	Red meat (roasted, grilled, stewed, à Milanese, minced, meatball and dry)	0.382	
	Bergamot, mango, watermelon, grape	0.366	
	Sugar	0.361	
	Margarine	0.312	
	French fries or shoestring potatoes and fried polenta	0.301	
DP II (Fruits and vegetables)			
	Chayote, eggplant and zucchini	0.516	7.843
	Fruit juice	0.482	
	Papaya	0.478	
	Vegetable soup	0.464	
	Broccoli, cauliflower and cabbage	0.438	
	Brown bread	0.375	
	Butter	0.360	
	Watercress, lettuce, spinach, rocket and kale	0.333	
	Banana, apple and orange	0.329	
	Whole-grain rice	0.325	
DP III (Common Brazilian)			
	Cassava, yam and farofa	0.549	5,539
	Chicken (crumbed, fried, stewed, grilled and baked)	0.494	
	Egg (fried and boiled)	0.369	
	Fish (stewed, baked and fried)	0.353	
	Potato (boiled, baked and mashed)	0.335	
	Black beans	0.323	
	Polenta (baked)	0.316	
Total of explained variance			21,71%

KMO: 0.590; Bartlett sphericity test: <0.001; DP: dietary pattern.

Table 3 shows that women in the highest quintile for the regional dietary pattern had the highest mean values for BMI and WC. Women in the lowest quintile for the common Brazilian dietary pattern had the highest mean values for WC. We also observe an association between the total of meals per day and the presence of a mid-afternoon snack with BMI and WC, and women who consumed five or more meals per day had the lowest mean values for BMI and WC, as well as those women who reported having a mid-afternoon snack.

**Table 3:** Mean and standard deviation of body mass index and waist circumference according to diary meals intake and the frequency of food consumption of menopausal women of Caxias do Sul, Brazil

Variable		N (%)	BMI Mean (sd)	*p-value*	WC Mean (sd)	*p-value*
Dietary Patterns						
DP Regional				0.027*		0.013*
	Quintile 1	62 (19.7)	28.39 (4.10)		87.22 (10.81)	
	Quintile 2	63 (20.0)	29.16 (4.58)		88.43 (11.49)	
	Quintile 3	63 (20.0)	28.34 (5.08)		88.54 (13.46)	
	Quintile 4	63 (20.0)	29.17 (4.91)		88.82 (9.96)	
	Quintile 5	63 (20.0)	30.50 (4.98)		92.62 (10.20)	
DP Fruits and Vegetables				0.894*		0.169*
	Quintile 1	63 (20.0)	28.41 (4.52)		89.23 (11.88)	
	Quintile 2	64 (20.3)	29.47 (4.78)		89.82 (12.21)	
	Quintile 3	62 (19.7)	29.74 (4.63)		90.33 (9.77)	
	Quintile 4	64 (20.3)	29.85 (5.40)		90.43 (12.20)	
	Quintile 5	61 (19.4)	28.05 (4.33)		85.73 (9.92)	
DP Common Brazilian				0.082*		0.041*
	Quintile 1	62 (19.7)	29.98 (4.03)		91.62 (10.92)	
	Quintile 2	64 (20.3)	29.18 (4.21)		89.59 (11.59)	
	Quintile 3	62 (19.7)	29.39 (4.72)		89.56 (10.48)	
	Quintile 4	63 (20.0)	28.27 (4.85)		86.30 (11.28)	
	Quintile 5	63 (20.0)	28.77 (5.83)		88.63 (12.01)	
Total meals				0.038*		0.040*
	≤4 meals	103 (32.2)	29.89 (4.73)		90.98 (11.20)	
	≥5 meals	216 (67.5)	28.70 (4.77)		88.19 (11.29)	
Mid-afternoon snack				0.005		0.002
	Yes	44 (13.8)	30.94 (4.95)		94.04 (10.82)	
	No	276 (86.3)	28.79 (4.69)		88.33 (11.21)	

*p-*value – t-test or ANOVA; sd: standard deviation; BMI: body mass index; WC: waist circumference.

After adjustment, the highest adherence to the regional dietary pattern was associated with the outcomes. The increase of 1 in the regional pattern’s consumption score resulted in an increase of 0.56 kg/m² in the BMI and 1.28 cm in WC. On the other hand, consuming five or more meals per day was inversely associated with BMI and WC and the consumption of a mid-afternoon snack (Table 4).

**Table 4:** Adjusted regression coefficients (β) and confidence intervals (95% CI) to estimate the association between dietary patterns, diary meals intake characteristics and anthropometric measurements of menopausal women of Caxias do Sul, Brazil

Variables	BMI				WC			
	β crude* (95%CI)	*p-value*	β adjusted* (95%CI)	*p-value*	β crude* (95%CI)	*p-value*	β ajusted** (95%CI)	*p-value*
DP Regional	0.42 (0.04; 0.79)	0.027	0.56 (0.03; 1.08)	0.037	1.12 (0.23; 2.00)	0.013	1.28 (0.17; 2.55)	0.047
DP Fruits and Vegetables	-0.02 (-0.40; 0.35)	0.895	0.10 (-0.42; 0.63)	0.703	-0.62 (- 1.51; 0.26)	0.171	-0.31 (-1.56; 0.92)	0.615
DP Common Brazilian	-0.33 (-0.70; 0.04)	0.081	-0.39 (-0.92; 0.13)	0.142	-0.92 (- 1.81; -0.03)	0.041	-1.21 (- 2.47; 0.03)	0.056
≥ 5 meals/day	-0.21 (-1.29;0.86)	0.696	-1.18 (-2.30; -0.05)	0.041	-0.57 (-3.12; 1.97)	0.659	-2.77 (-5.41; -0.13)	0.039
Mid-afternoon snack	-2.15 (-3.66; -0.64)	0.005	-2.16 (- 3.66; -0.65)	0.005	-5.71 (-9.27; -2.14)	0.002	-5.76 (-9.29; -2.23)	0.001

* Adjusted to marital status.

** Adjusted to marital status and physical activity.

BMI: body mass index; WC: waist circumference; DP: dietary pattern; CI: confidence interval.

Finally, there was an inverse association between the consumption of a mid-afternoon snack and the adherence to a regional dietary pattern (β = –0.63 [CI95% = –1.07 to –0.18], p = 0.006) and between the consumption of five or more meals per day (β = –0.51 [CI95% = –0.84 to –0.18], p = 0.002). There was a positive association between the consumption of five or more meals per day and the fruits and vegetables dietary pattern (β = 0.35 [CI95% = 0.02-0.69], p = 0.034) (data was not tabulated).

## DISCUSSION

This study contributes to the scientific literature on understanding the relation of eating habits and excessive weight gain on aging, primarily by showing the association of a regional eating pattern, composed both by traditional foods, considered healthy by food guidelines and by processed foods understood as a western influence on eating habits, associated with higher BMI and WC. This finding highlights the complexity of individual eating habits, mainly of older people, who, on the one hand, still keep their culturally traditional eating habits but also include more modern and globalized eating habits. As far as it is known, this study is the first to associate dietary patterns and the number of meals per day, showing that these two eating habits are associated and may together contribute to the etiology of obesity internationally.

It was possible to identify three dietary patterns in menopausal women living in the southern region of Brazil. The regional dietary pattern was characterized by consuming foods commonly found in the region called “Serra Gaúcha” (highlands in the State of Rio Grande do Sul, Brazil). This pattern denoted the influence of western dietary patterns into the habits inherited from the Italian immigrants who colonized this region at the end of the 19^th^ Century, presented as the mixing of traditional foods such as pasta, bread, meats, vegetables, and fruits commonly found in this region (grapes and tangerine) with processed foods such as margarine, sugary drinks (soda), fried foods, and pastries. Other studies with menopausal women had found similar traditional dietary patterns, including a mix of healthy and industrialized foods in different countries (22,23).

The adherence to this regional pattern was directly related to BMI and WC in our study. These associations can be explained, in part, by the high energy density foods included in this pattern, mostly rich in carbohydrates with a high glycemic index, and fat, such as pasta, bread, jam, sugar, meat and fried foods; as well as ultra-processed foods, such as soda and margarine. Studies have linked rising obesity rates to the consumption of an increasingly energy-dense diet. Energy-dense foods, often high in refined grains, added sugars, and added fats, are palatable, inexpensive, and convenient. However, they have been associated with increased energy intake and poor diet quality (24). At the same time, research ranging from laboratory-based to prospective epidemiological studies and experimental evidence suggest that ultra-processed foods affect cardiometabolic health. Key biological pathways include altered serum lipid concentrations, modified gut microbiota, obesity, inflammation, oxidative stress, insulin resistance, and hypertension (25). Other studies done on a similar dietary pattern found a positive correlation with obesity. Papavagelis and cols. (2018), in Tehran, have found a dietary pattern with high consumption of potatoes and red meat and low consumption of nuts, coffee and tea, similar to our regional pattern, positively associated with general and abdominal obesity in adult women (8).

In addition, the regional dietary pattern was inversely associated with the number of meals per day. We have not been able to find studies that investigated the association between dietary patterns and the number of meals per day. However, it is plausible to explain this finding because the foods included in the regional pattern are also energetically dense and seem to provide more extended periods of satiety, culminating in a lower number of meals per day (26).

Fruits and vegetables dietary pattern was composed mainly of these foods and also whole grains. Other studies found similar dietary patterns (22,23,27). Heidari and cols. (2018) have named a similar pattern as “healthy”. It was composed of fruits, vegetables, seeds, beans, fish, seafood, whole grains, liquid fats, olive oil, olives, and low sodium intake (23). Hoffmann and cols. (2015) have also identified an eating pattern called “fruits and vegetables” composed only of these foods (28), similarly to our results. This dietary pattern was positively correlated to consuming five or more meals per day. This finding may be explained by the fact that this healthy dietary pattern is composed of foods with a lower energy density, thus resulting in a higher number of meals per day, due to the low feeling of satiety that these foods provide (27). In this sense, with the quality of the diet, Mills and cols. (2011) have already demonstrated that the ingestion of fruits, vegetables, whole grains, dietary fiber, and dairy increases along with the number of meals consumed per day (14).

The common Brazilian dietary pattern was composed of foods usually consumed by Brazilians. Other studies conducted in the southern parts of Brazil have found similar results. Hoffmann and cols. (2015) have found a similar pattern called “Brazilian” composed of rice, beans, and milk (28). Ternus and cols. (2019) have found in a sample of women between 20 and 69 years old a similar eating pattern, mostly composed of rice and beans (27). In this study, there is no significant association between this pattern and outcomes or eating frequency.

In addition to identifying eating patterns, the results of this study also point out that an increase in the number of meals per day and adding a snack in the middle of the afternoon seem to result in lower BMI and WC in menopausal women, despite inconclusive evidence found in systematic revisions on the subject (12,13). A transversal study using data from NHANES 2003-2012 has also found a positive correlation between the number of meals per day and abdominal obesity in US men and women ≥20 year of age (29). Another transversal study has investigated the correlation between food intake frequency and BMI using data from INTERMAP, including men and women from 40 to 59 years old, from China, Japan, United Kingdom and the United States, and it has shown that those who had six or more had a lower BMI (27.3 vs. 29 kg/m²) compared to individuals that had less than four meals per day (30). The biological mechanism involved with the increase of the number of meals per day and the decrease in body weight is shown to increase metabolism (31), control the appetite and food intake (32,33), and an improvement in glucose and insulin control (34) which, together, seem to be able to reduce body weight due to the increase in meals per day.

In addition, women who reported habitually having a mid-afternoon snack have presented lower anthropometric measures. However, this finding might be only highlighting the importance of a mid-afternoon snack concerning the total caloric intake in a day (35) because those women who said they had included a mid-afternoon snack in their days had possibly ingested less energy than those who had fewer meals per day, which in turn would result in lower BMI and WC numbers.

This study has some limitations. First, the information bias is a limitation inherent to the foods reported. Second, the methodology of identifying dietary patterns involves choices that might be subjective to the researcher, for example, the choice of the criteria with which to group foods and the type of rotation used in the factorial analysis. Different choices could lead to different dietary patterns. Third, the KMO value found in this study was lower than the ideal indicated in the literature. Nevertheless, the analysis of it is possible to consider the coherence of patterns found compared to the existing literature and the biological plausibility in their association with the investigated endpoints, both the anthropometric figures and the frequency of food consumption. Fourth, the women are participants of a project that improves healthy habits, such as physical activity. Thus these women could be healthier than the general population.

In conclusion, three dietary patterns were identified in this population: (i) regional, (ii) fruits and vegetables, and (iii) common Brazilian. The regional dietary pattern, composed of culturally habitual food consumed in the region and industrialized food, was associated with higher BMI and WC. Furthermore, this dietary pattern was also associated with a decrease in the number of meals per day, showing that women who adopted this dietary pattern also had fewer meals per day and had higher anthropometric measures. On the other hand, adopting the dietary pattern of fruits and vegetables, formed by foods considered healthy and low in calories, was associated with an increase in the number of meals per day, which was associated with lower anthropometric measures. Finally, the consumption of five or more meals per day is inversely associated with BMI and waist circumference and the consumption of a mid-afternoon snack.
